# The brain as an HIV reservoir: Recent findings using autopsy tissues from people with HIV

**DOI:** 10.1371/journal.ppat.1014446

**Published:** 2026-07-29

**Authors:** Rajnish S. Dave, Ayo A. Oludipe, Elizabeth C. Pasipanodya, Seth Sherman, Steffanie H. Wilson, Dana H. Gabuzda, Melissa J. Churchill, Thomas A. Angelovich, Robert J. Gorelick, Sara Gianella, Antoine Chaillon, Jerel Adam Fields, Susan Morgello, Benjamin B. Gelman, David J. Moore, Elyse J. Singer, Beau M. Ances, Howard S. Fox

**Affiliations:** 1 Department of Neurological Sciences, University of Nebraska Medical Center, Omaha, Nebraska, United States of America; 2 Department of Pathology, Microbiology, and Immunology, University of Nebraska Medical Center, Omaha, Nebraska, United States of America; 3 The Emmes Company LLC, Rockville, Maryland, United States of America; 4 Dana-Farber Cancer Institute and Harvard Medical School, Boston, Maryland, United States of America; 5 ATRACT Research Centre, School of Health and Biomedical Sciences, Royal Melbourne Institute of Technology (RMIT) University, Melbourne, Victoria, Australia; 6 AIDS and Cancer Virus Program, Frederick National Laboratory for Cancer Research, Frederick, Maryland, United States of America; 7 Department of Internal Medicine, University of California, San Diego, La Jolla, California, United States of America; 8 Department of Psychiatry, University of California, San Diego, La Jolla, California, United States of America; 9 Department of Neurology, Icahn School of Medicine at Mount Sinai, New York, United States of America; 10 Department of Pathology, University of Texas Medical Branch, Galveston, Texas, United States of America; 11 Department of Neurology, University of California, Los Angeles, Los Angeles, California, United States of America; 12 Department of Neurology, Washington University School of Medicine, St. Louis, Missouri, United States of America; University of North Carolina at Chapel Hill, UNITED STATES OF AMERICA

## Abstract

HIV persistence within anatomical reservoirs remains the primary barrier to achieving an HIV cure. While antiretroviral therapy effectively suppresses plasma viremia, it does not eliminate integrated proviral genomes that persist in long-lived cellular compartments. The central nervous system (CNS) is a clinically important HIV reservoir, characterized by immune privilege and the persistence of tissue-resident infection despite effective antiretroviral therapy (ART). Evidence from postmortem studies reveals that HIV DNA, RNA, and even intact replication-competent proviruses remain detectable in brain tissue from virally suppressed people with HIV. Evidence derived primarily from *in situ* approaches and viable-cell studies supports myeloid-lineage reservoirs, particularly microglia and CNS-associated macrophages, as key cellular sources of persistence, while the extent and biological relevance of astrocyte infection remains debated. These reservoirs exhibit transcriptional activity and are associated with chronic neuroinflammation, which may contribute to HIV-associated neurocognitive disorders, despite systemic viral suppression. Here, we synthesize recent findings from autopsy brain studies, including work enabled by major biorepositories, such as the National NeuroHIV Tissue Consortium and rapid-autopsy programs, including the Last Gift, both of which are essential for studying HIV reservoirs in the CNS. We summarize methodologies for detecting and characterizing HIV in brain tissue, highlight heterogeneous patterns of regional distribution and compartmentalization, and review emerging links between CNS persistence and neuroinflammation. We conclude with priorities for harmonized tissue processing, multi-modal single-cell and spatial profiling, and coordinated cross-cohort analyses to clarify the contribution of CNS reservoirs to neuroHIV pathogenesis and systemic rebound.

## Introduction

Despite the success of antiretroviral therapy (ART) in suppressing plasma viremia, HIV persists as integrated proviral DNA in long-lived cellular compartments and tissues, thereby enabling viral rebound if treatment is interrupted and contributing to chronic immune activation even during suppression. HIV reservoirs are widely recognized as the major obstacle to viral eradication and remain the central challenge to achieving a cure. Although ART effectively suppresses plasma viremia and prevents disease progression, it does not eliminate integrated proviral genomes that persist within long-lived cellular and tissue compartments. While resting memory CD4⁺ T cells remain the best-characterized reservoir [1,2], tissue-resident and immune-specialized compartments are likely governed by distinct biological constraints, require tailored measurement approaches, and may differ in their relevance to clinical outcomes. Additional reservoirs exist in lymphoid tissues, gut-associated lymphoid tissue, and immune-privileged sites such as the central nervous system (CNS), each of which may be governed by distinct mechanisms of anatomical and immunological factors that influence viral persistence [3]. Crucially, these latent reservoirs are refractory to ART and largely evade host immune surveillance, resulting in viral rebound following treatment interruption in most people with HIV (PWH) [4].

Beyond viral rebound, accumulating evidence indicates that HIV reservoirs contribute to persistent low-level viral transcription and viral protein production despite effective plasma viral suppression, thereby sustaining chronic immune activation and systemic inflammation [5,6]. These ongoing inflammatory processes are strongly associated with an increased risk of non-AIDS comorbidities, including cardiovascular disease, neurocognitive impairment, and accelerated biological aging among PWH [7,8]. Accordingly, defining the cellular and anatomical distribution of HIV reservoirs, elucidating the molecular mechanisms that establish and maintain latency, and developing strategies to eliminate or durably silence these reservoirs across all tissues constitute critical steps toward achieving a functional cure or complete viral eradication.

Among these, the CNS represents a critical and distinct reservoir for HIV persistence. It was recognized as a site of HIV involvement early in the epidemic [9,10], with viral entry occurring shortly after systemic acquisition through the trafficking of infected leukocytes. Once established, HIV persists in long-lived CNS-resident cells, most notably cells of the myeloid lineage, including perivascular macrophages and microglia, and potentially in other cell types such as astrocytes [11,12]. The CNS is an immune-privileged site, and the blood–brain barrier restricts both immune surveillance and penetration of many antiretroviral drugs, thereby facilitating viral persistence despite suppressive ART [13]. Of note, HIV persistence within CNS parenchyma cannot be inferred from cerebrospinal fluid (CSF) alone. CSF is synthesized primarily by the choroid plexus, through which immune cells can traffic, and only a minor fraction represents brain interstitial fluid [14]. On the other hand, brain tissue provides direct access to the cellular and anatomical reservoirs that underlie CNS infection.

Viral activity in the CNS is linked to neuroinflammation and can contribute to functional cognitive deficits, and as with other causes of cognitive dysfunction, is referred to generally as neurocognitive impairment (NCI). The term neuroHIV is used to refer to CNS disorders resulting from HIV, which includes terms such as HIV-associated neurocognitive disorders (HAND) [15] and HIV-associated brain injury (HABI) [16]. While HIV infection leads to neuroHIV, in the current era of suppressive ART the cause of cognitive impairment in PWH remains unknown [15–18]. Together this underscores the need to specifically target the brain in curative strategies. Because much of what is known about HIV persistence in the brain derives from post-mortem tissue studies, it is essential to characterize the biorepositories that support this research and to understand their use in research on HIV in the brain.

### Human brain biorepositories supporting NeuroHIV research

Several biobanks and research programs have substantially advanced our understanding of HIV persistence in the brain. Two have contributed substantially to the study of HIV in the brain: The National NeuroHIV Tissue Consortium (NNTC) and Last Gift (LG). The NNTC is a federated system of five biorepositories that together are distinguished by a large participant cohort and extensive, longitudinal in-life clinical characterization that provides data associated with most tissues collected. Thus far, 3,567 participants, including 3,094 PWH, have been enrolled in the NNTC, and 1,100 brains (along with other vital organs) have been donated to the biobank, alongside tens of thousands of longitudinal plasma and CSF samples. The NNTC collects extensive baseline and longitudinal data to inform analyses, and biofluids are available for requestors (S1 Table). Participants or legal proxies consent to autopsy, and the NNTC collects post-mortem brain and peripheral tissue, performs pathological assessments, and banks frozen and fixed tissue, providing investigators with a wide-ranging framework for examining the effects of HIV on the CNS over time (S2 Table). The NNTC collects neurological, neurocognitive, psychiatric, and clinical laboratory data during life. Because the time and frequency of viral load measurements vary across participants, two related but distinct operational definitions of viral suppression were instituted in the NNTC cohort (S3 Table). Forty-three participants met Criterion 1, requiring ≥5 years of observation, with ≥4 undetectable viral loads in the last 5 years, and either one undetectable value within 12 months of death or two within 24 months. Seventy-five participants met Criterion 2, requiring ≥2 years of observation and at least one undetectable viral load within 6 months of death. Since these criteria are overlapping, 33 participants met both criteria, for a total of 85 participants in the NNTC suppressed autopsy cohort. Information on these NNTC participants with brain autopsy specimens is presented in S4 Table. Given the effectiveness of contemporary ART regimens, this suppressed autopsy cohort is expected to grow, as currently 71% of active NNTC participants currently have viral loads that are below the limits of detection of current standard assays (<20 copies/mL).

In addition to the site-collected central data, experimental data generated by investigators are returned to the consortium and entered into a case-linked database [19]. These attributes established NNTC as one of the most widely utilized and valuable resources for the study of the effect of HIV on the CNS, including studies on HIV persistence in the brain. Investigators can request specimens and data from the NNTC upon approval of their request, which can be made at http://nntc.org/. To date, the NNTC has received 1,038 requests, resulting in the shipment of 29,461 specimens, along with linked clinical data and metadata.

A complementary effort closely integrated with the NNTC is the LG study, which works in collaboration with studies on PWH at the San Diego NNTC site, the California NeuroHIV Tissue Consortium (CNTN). Complementing the NNTC, the LG program, a rapid-autopsy cohort designed to investigate HIV persistence at the end of life, has provided unique insights into CNS reservoirs through near-death sampling of individuals maintained on suppressive ART until death. In addition to achieving short postmortem intervals, participants undergo intensive prospective clinical and virologic follow-up, with plasma HIV-1 RNA measurements frequently obtained within days, and sometimes hours, of death. The average time from last plasma viral load measurement to death for LG participants is 20 days, with a median of just 6 days. Intracardiac fluid is collected at autopsy to enable additional virologic characterization. Together, these features provide an unusually detailed view of reservoir biology in the context of virologic status immediately preceding death. Upon death, the LG team conducts a rapid research autopsy enabling the collection of high-quality brain and peripheral tissues with minimal post-mortem interval. The time from participant death to completion of the autopsy by the LG team averages 6.8 hours (with a median of 6.05 hours). This approach preserves labile viral and host signals, maximizing RNA integrity and cell viability, thereby providing unique opportunities to study HIV persistence, transcriptional activity, and reservoir dynamics that are not possible with standard autopsy protocols. Because the rapid-autopsy model is highly resource-intensive, the LG cohort is necessarily smaller than the broader NNTC; however, the complementary strengths of these programs, combining deep longitudinal characterization with near-death assessments and rapid tissue collection, provide a powerful framework for studying CNS HIV reservoirs.

Both of these cohorts rely on participants who consent to brain and organ donation at death for research purposes. When the NNTC was established, the program drew on a substantial literature documenting barriers to organ donation within minority communities, and great consideration was given to these issues within the broader context of palliative care. Particular attention was given to developing ethically appropriate approaches for engaging seriously ill individuals in discussions about end-of-life tissue donation. The LG initiative has further expanded this by examining the ethical, social, and emotional dimensions of end-of-life HIV research through a deeply participant-centered model. This work incorporates the perspectives of healthcare workers and research staff, highlighting participant altruism, emotional challenges, and the central role of community engagement in building trust and supporting participants and families with dignity, consent, and respect [20,21]. Together, this has demonstrated a broad willingness among PWH and their families to participate in observational and interventional cure-related research at the end of life and underscores the value participants place on contributing to scientific progress [22–24].

Although many earlier studies examined brain tissue from PWH before the widespread availability of highly effective suppressive ART, several reports in the past five years have focused specifically on individuals who were virally suppressed at the time of death. In the sections below, we indicate whether samples originated from an NNTC participant, an LG participant, or other sources.

As described above, data from investigator-performed studies generated from specimens from NNTC participants must be provided back to the consortium. We have now compiled all available data on quantitative reservoir studies on brains from NNTC’s virally-suppressed cohorts in S5 Table. Information on quantitative proviral loads from brain and lymphoid tissues from NNTC and LG participants are given in Table 1.

**Table 1:** Proviral HIV loads from brain and lymphoid tissues in suppressed participants.

**Last Gift**					
**Tissue**	**# Participants**	**# Samples**	**Detectable**	**% detectable**	**Median [IQR]**
CNS	28	258	192	74.4	4.08 [15.04]
Lymphoid	28	64	63	98.4	50.8 [90.1]
**NNTC**					
**Tissue**	**# Participants**	**# Samples**	**Detectable**	**% detectable**	**Median [IQR]**
CNS	38	165	93	56.4	2.53 [20.53]
Lymphoid	21	23	23	100	85.55 [146.37]

IQR, intraquartile range.

The goal of this review is to synthesize the published literature that utilizes autopsy brain tissue to examine the CNS as a potential reservoir. We summarize current findings and state of knowledge regarding HIV persistence in the brain, drawing on data from multiple cohorts and tissue sources. We also highlight important directions for future research to fill key gaps in the field to advance our understanding of CNS HIV reservoirs.

## Review of current literature

### Ethics statement

Studies on post-mortem samples are not considered human subjects research. The NNTC and LG operate under IRB approval at their respective institutions.

### Identifying HIV-positive cells and characterizing viral persistence

Detection and quantification of cells harboring HIV nucleic acids, together with characterization of viral genomes and transcriptional activity, are key to determining whether HIV persists in the CNS, expresses viral gene products, and retains the potential to produce replication-competent virus. A range of complementary laboratory techniques has been developed for HIV reservoir studies, each with distinct strengths and limitations [25–28]. Importantly, detection of HIV nucleic acids or proteins does not by itself establish ongoing replication or pathogenic relevance, underscoring the need to integrate multiple complementary assays. We note that for many of the techniques, the lower limit of detection may not be known or may differ between labs performing the assay. In the studies described below, the criteria used to define the populations under study, the criteria for suppression (which often differ from the above NNTC criteria), and their findings are summarized in Table 2.

**Table 2:** Summary of brain viral reservoir studies discussed in this manuscript.

Cohort and Reference	Participant characteristics	Study’s criteria for viral suppression	Tissues		Techniques	Major finding(s)
			CNS	Other		
NNTC	PWH and PWoH	PWH on suppressive ART with undetectable plasma VL	Frontal white matter, Basal Ganglia, cerebellum	N/A	ddPCR, IPDA	Frontalwhite matter is a major brain reservoir of intact, potentially replication competent HIV DNA
Angelovich and colleagues (2023), PMC10914117						
NDRI	PWH	Well-controlled PWH with plasma VL < 75 copies/mL at the last test (7 months prior to death); post-mortem plasma/serum undetectable	Frontal lobe, Occipital lobe, Parietal lobe	Lung, Lymph nodes, Colon and Blood cells	Single-genome SMRT sequencing	Complete compartmentalization of brain HIV env sequences, with exclusion of CXCR4-using variants from brain tissue
Brese and colleagues, PMC7281851						
NNTC	PWH and PWoH	Suppressed PWH had undetectable plasma VL while on ART	Frontal cortex	N/A	IPDA	Cell activation persists in the brain of virally suppressed PWH and is associated with HIV DNA
Byrnes and colleagues (2024), PMC12135078						
Last Gift	PWH with terminal non-HIV conditions	Suppressed participants had undetectable plasma VL (<20 copies/mL) on ART until death. Participants who stopped ART had last plasma VLs of 280 and 48,000 copies/mL	Frontal cortex, Occipital cortex	Lymph nodes, Blood plasma, Cardiac serum, Gut, Kidney, Liver, Lymphoid tissue, Pancreas, PBMC, Pericardial adipose tissue, Genital tract	ddPCR; single-genome amplification	Intact HIV DNA was detected across all deep tissues. Viral exchanges occurred both within the CNS and between CNS and blood. During ART interruption, large clonal intact HIV RNA populations in blood were found in multiple tissues, indicating that blood replenishes virus in tissues
Chaillon and colleagues (2020), PMC7108926						
NNTC	PWH with HIVE and virally suppressed PWH on ART	Suppressed PWH had plasma VL < 50 copies/mL while on stable ART prior to death.	Frontal cortex, Corpus Callosum, Basal Ganglia	N/A	ddPCR, qPCR	HIV DNA in the brain was detected more frequently by ddPCR than by qPCR
Chung and colleagues (2022), PMC8754137						
NNTC	PWH and PWoH	Suppressed PWH on ART, defined as undetectable plasma VL on standard assays	Frontal lobe deep white matter	Lymphoid tissue (Spleen, Lymph node, or Gut-associated lymphoid tissue)	IPDA	8 of 10 viremic and 6 of 9 virally suppressed PWH also harbored intact proviruses in the CNS (4.63 vs 12.7 intact copies/10^6 cells).
Cochrane and colleagues (2022), PMC9489665						
NNTC	ART-treated PWH classified as undetectable, low, or high systemic viral replication	Categorized by plasma VL (undetectable, low, high)	Frontal cortex, Hippocampus	Spleen, Liver, Bone marrow, Urethra, Vagina, Heart, Gut	Multiplex imaging, RNAscope, confocal	HIV DNA, RNA, and proteins were detected in CNS myeloid cells even in individuals with undetectable plasma VL, supporting persistence of CNS reservoirs during ART.
Donoso and colleagues (2022), PMC9367788						
NNTC	Virally suppressed PWH on long-term ART	Suppressed PWH had undetectable plasma VL and were on ≥3 ART drugs for at least two years, with their last plasma VL within 12 months of death being undetectable	Frontal lobe white matter	N/A	IPDA, HIV gag DNA/RNA assays	PWH exhibited broad upregulation of inflammation and stress-related genes and downregulation of oligodendrocyte/white-matter genes, suggesting neuroinflammatory activity even under plasma viral suppression.
Gabuzda and colleagues (2023), PMC10142371						
NNTC	ART-treated PWH with undetectable plasma VL	Documented undetectable plasma VL on ART;	Frontal lobe white matter, Basal Ganglia and Corpus Callosum	N/A	RNAscope and DNAscope ISH; immune-histochemistry with cell markers	HIV from virally suppressed cases in macrophages and microglia, but not astrocytes
Ko and colleagues (2019), PMC6391194						
NNTC	Non-virally suppressed and virally suppressed PWH	<50 copies/mL; on ART ≥ 2 years; some participants discontinued ART prior to death	Frontal cortex	N/A	QIAcuity 5-plex RT-dPCR	HIV transcription persists in the brains of virally suppressed PWH, with multiple HIV RNA species detected in frontal cortex and microglia; transcriptional activity correlated with total HIV DNA levels.
Jamal Eddine and colleagues (2024), PMC11335163						
NNTC	PWH with uncontrolled plasma virus (HIV+), undetectable plasma virus (HIV + /ART), and PWoH (HIV-)	PWH subgroup classified as suppressed while on ART (undetectable plasma VL)	Cerebral cortex	N/A	ddPCR	ART did not suppress viral burden in the brain, regardless of ART component regimen, the duration of therapy, and its interruption.
Mohammadzadeh and colleagues (2021), PMC8669467						
NNTC	PWH with or without ART	Participants 3 and 4 were considered virally suppressed based on ART treatment and undetectable plasma VL on ART.	Frontal lobe, Subventricular zone, Occipital lobe	N/A	ddPCR & IPDA	A distinct HIV brain reservoir in microglia for all individuals. Intact proviruses detected in only one.
Nuhn and colleagues (2025), PMC12030925						
NNTC	PWH with no evidence of CNS opportunistic diseases. Undetectable plasma VL at last visit prior to death.	NNTC donors on suppressive ARTwith undetectable plasma VL at last clinical visit	Frontal cortex, Occipital cortex, Basal Ganglia	Lymph node, Spleen	ddPCR, proviral sequencing	Differential distribution of HIV reservoirs across brain regions which was lower than that in lymphoid tissues.
Oliveira and colleagues (2023), PMC10308944						
NNTC	PWH with cancer pathology and undetectable plasma VL at last visit before autopsy	On suppressive ART with undetectable plasma VL near death	Cerebellum, Frontal cortex, Basal ganglia, Meninges, Occipital lobe, Temporal lobe	Liver, Lymph node (tumor), Lung, Spleen, Colon, Kidney, Aorta, Pancreas	single genome sequencing	Brain is a sanctuary site for wild type HIV during cART. There is sequence compartmentalization with distinct evolution in CNS and LN.
Rose and colleagues (2016), PMC5044847						
Last Gift	Virally suppressed PWH	Plasma VL levels below the limit of detection	Dorsolateral prefrontal cortex (DLPFC) microglia	N/A	scRNA-seq, scATAC-seq	Microglia from virally suppressed PWH showed persistent interferon/ inflammatory transcriptional programs and altered chromatin accessibility, indicating an activated CNS immune environment despite long-term ART.
Schlachetzki and colleagues (2024), PMC11282357						
MGH Autopsy	PWH on suppressive ART	Plasma VL below the limit of detection on ART	Frontal cortex, Occipital cortex, Basal Ganglia, Cerebellum	Spleen, Liver, Lymph nodes, Kidney, Colon	Near-full-length proviral sequencing, IPDA	Intact HIV proviruses persist in multiple brain regions of virallysuppressed individuals, demonstrating a durable CNS reservoir during long-term ART.
Sun and colleagues (2023), PMC10631759						
Last Gift	PWH on durable ART,virally suppressed at end of life	Plasma VL below detection while on long-term ART	Parietal cortex, Basal Ganglia	PBMCs, Lymph nodes, Spleen	ddPCR, viral outgrowth	Replication-competent HIV persisted in brain microglia of virally suppressed individuals, demonstrating that microglia constitute a long-lived, ART-resistant CNS reservoir.
Tang and colleagues (2023), PMC10266791						
NNTC	PWH on long-term ART with systemic viral suppression	Clinically documented systemic viral suppression.	Frontal cortex and subcortical areas	N/A	Confocal microscopy, RNAscope, HIV-p24 staining, IHC	Astrocytes contained integrated HIV DNA and were capable of transferring HIV to myeloid cells, indicating a persistent CNS reservoir despite ART.
Valdebenito and colleagues (2021), PMC11102126						

N/A, not assessed; VL, viral load.

Collectively, the studies summarized in Table 2 support an overarching conclusion that the CNS constitutes a durable HIV sanctuary during suppressive ART, with HIV DNA, RNA, and in several cases intact or replication-competent virus detected across multiple brain regions, most prominently frontal cortex, white matter, and basal ganglia [29–33]. CNS myeloid cells, microglia and macrophages, emerge as the dominant cellular reservoirs, while astrocytes can harbor proviral DNA and may facilitate viral persistence without robust productive infection [34,35]. However, reported variability in reservoir size, proviral integrity, transcriptional activity, and CNS vs peripheral compartmentalization reflects substantial differences in the participants’ conditions, including ART duration, comorbid conditions, peri-death ART interruption, and inconsistent definitions of viral suppression [31,36,37]. Methodological heterogeneity further contributes to divergent conclusions. Bulk ddPCR and qPCR maximize sensitivity for total HIV DNA but lack resolution of proviral integrity, whereas intact proviral DNA assay (IPDA) and near-full-length sequencing are limited by DNA fragmentation. *In situ* and single-cell approaches provide cellular localization and transcriptional context but often underestimate low-abundance infection due to RNA degradation and limited tissue depth [38–40]. Given the size of the brain and small regions taken for study, sampling bias exists for all methods. Together, these technical and biological constraints underscore that apparent discrepancies across studies largely reflect differences in sampling, assays, and clinical context rather than fundamental disagreement, reinforcing the CNS as a distinct HIV reservoir during ART.

### Quantification of HIV in brains from virally suppressed PWH

Many studies using autopsy brain tissues from PWH have focused on detecting and quantifying HIV DNA and RNA in the CNS using PCR-based amplification methods. By amplifying, and in some cases sequencing, regions of HIV DNA and/or RNA, several groups have quantified proviral burden in the brain using the ddPCR, a highly sensitive technique capable of detecting low-level HIV nucleic acids, to evaluate the presence and distribution of HIV in brain tissue from virally suppressed individuals.

Mohammadzadeh and colleagues [41] analyzed autopsy brain samples from NNTC participants and other sources, assessing both HIV DNA and RNA. They found detectable HIV DNA in the cerebral cortex of all four virally suppressed individuals, as well as in all eight viremic PWH, at comparable levels between the two groups. HIV RNA was also detected in all four virally suppressed individuals and in seven of the eight viremic individuals, again at similar levels. Notably, virally suppressed PWH exhibited significantly higher levels of integrated HIV proviral DNA compared with viremic PWH.

Chung and colleagues [42] examined brain tissue from 27 NNTC participants, 16 who were virally suppressed and 11 viremic donors with HIV encephalitis (HIVE). Three CNS regions were examined for the presence of HIV DNA: the basal ganglia, frontal white matter, and corpus callosum. HIV DNA was detected in 5 of the 16 suppressed participants. In contrast, HIV DNA was detected in brain specimens from all 11 non-suppressed participants with HIVE.

Paired autopsy brain and lymphoid tissues from 63 virally suppressed NNTC participants were studied by Oliveira and colleagues [37]. Detection frequencies of HIV DNA were 70% (44 of 63) in the frontal cortex, 70% (41 of 59) in the basal ganglia, and 55% (31 of 56) in the occipital cortex, with significantly lower levels observed in the occipital cortex compared with the other regions. HIV DNA was detected in lymphoid tissue from all participants, underscoring the persistence of viral reservoirs outside the brain.

Quantification of HIV DNA has also been performed on brain regions from six LG participants, four of whom had undetectable plasma viral loads at, or immediately prior to death, in studies by Chaillon and colleagues [31] and Tang and colleagues [35]. HIV was detected in brain tissues from all four LG participants with undetectable plasma viral loads, as well as one of the two with viremia.

### Intact versus defective proviruses in the brain

The development of the IPDA, which identifies proviral sequences that are genetically intact and therefore more likely to be replication competent, enables discrimination between intact and defective HIV genomes in tissue-based reservoir studies [43]. Although IPDA provides an improved estimate of intact proviral burden, it remains a surrogate for replication competence and can generate false positive classifications [44].

Four studies from the Churchill group have applied IPDA and related quantitative approaches to brain tissue obtained from NNTC participants [38,45–47]. In the first, Cochrane and colleagues [45] detected HIV DNA in frontal lobe tissue from 12 virally suppressed and 18 viremic PWH, with no significant difference in total HIV DNA levels between groups. IPDA revealed that intact proviruses were present in 6 of 9 virallysuppressed and 8 of 10 viremic individuals, demonstrating that both groups harbored intact potentially replication-competent proviruses in the frontal lobe.

The second study, by Angelovich and colleagues [46], examined regional variation in intact proviral genomes across multiple brain regions. HIV *pol* DNA was detected in the frontal white matter, basal ganglia, and cerebellum in 16 virally suppressed and 21 viremic PWH. HIV *pol* DNA was detectable in all three brain regions, with lower levels in the cerebellum and basal ganglia compared with frontal white matter in both groups. Among those assessed for intact proviruses (8 of the 16 virally suppressed and 8 of the 21 viremic PWH), the frontal white matter contained intact proviruses in 6 of 8 virally suppressed and 6 of 8 viremic individuals. The basal ganglia contained intact proviruses in 2 of 8 virally suppressed and 4 of 6 viremic individuals. The cerebellum contained intact proviruses in 2 of 8 virally suppressed and 3 of 8 viremic individuals. Overall, the frontal white matter contained the highest levels of intact proviral DNA in both suppressed and viremic groups.

In a third study, Jamal Eddine and colleagues [47] evaluated paired autopsy brain and lymphoid tissues from 63 virally suppressed NNTC participants. The presence of HIV DNA was examined in the frontal cortex, basal ganglia, and occipital cortex. Detection frequencies were 70% (44 of 63) in the frontal cortex, 70% (41 of 59) in the basal ganglia, and 55% (31 of 56) in the occipital cortex, with significantly lower levels observed in the occipital cortex compared with the other regions. In contrast to the CNS findings, HIV DNA was detected in lymphoid tissue from all participants, underscoring the persistence of viral reservoirs outside the brain. Importantly, HIV RNA levels were strongly correlated with total HIV DNA, suggesting that transcriptional activity is tightly linked to the size of the brain reservoir.

In the most recent study from this group, Byrne and colleagues [38] assessed IPDA-defined measurements of intact and total proviruses in brain tissues from 18 virally suppressed and 17 viremic NNTC participants. As in the earlier work, no significant difference was observed in total HIV DNA between the two groups. This study then shifted focus to evaluating the relationship to neuroinflammation (discussed in the following section). Together, these studies indicate that intact and transcriptionally active proviruses are a persistent feature of the brain, even under long-term ART.

Other investigators have also applied IPDA to examine the HIV reservoir in autopsy brains from virally suppressed PWH. Gabuzda and colleagues [34] detected intact proviral genomes in 18 of 28 NNTC participants using IPDA. These researchers also assessed HIV DNA and RNA using *gag*-based PCR assays. They found that HIV DNA was detected in all 28 participants and HIV RNA in 26 of 28, confirming persistent viral genomes and transcriptional activity in brain tissue despite plasma viral suppression. While such IPDA studies have not been performed in the LG cohort, the study by Chaillon [31] found that 94% of *env* proviral sequences derived from the brains of 5 participants were intact.

Beyond IPDA, direct viral sequencing also provides strong evidence for the presence of intact proviruses in the brain. In a recent study examining postmortem brain tissue from three virallysuppressed individuals outside the NNTC and LG cohorts, Sun and colleagues [32] performed near-full-length proviral sequencing and identified intact, clonally related HIV genomes within the basal ganglia and periventricular white matter. These brain-derived sequences were phylogenetically linked to peripheral viral genomes, suggesting trafficking of infected cells or shared cellular ancestry or proviral trafficking mediated by cellular migration between brain and systemic reservoirs, both complementing and contrasting with the prior studies of viral compartmentalization. Overall, these findings demonstrate that the CNS can harbor genome-intact proviruses, clonally related to those in peripheral tissues, and support the possibility that infected cells migrate to and expand within the brain microenvironment.

Taken together, the above studies demonstrate that, despite effective systemic viral suppression, HIV can persist and show varying degrees of compartmentalization across brain regions and other tissues. This complexity underscores the difficulty of defining the boundaries and behavior of HIV reservoirs within the CNS.

### Brain cell types harboring HIV

Historically, in individuals with active viremia, numerous studies have demonstrated that cells of myeloid lineage, including microglia and perivascular macrophages, can express HIV RNA and protein within the CNS [48,49]. However, some studies have identified astrocyte infection in this pre-efficacious ART era. Churchill et al, using immunohistochemistry, *in situ* hybridization, and viral protein detection, reported extensive astrocyte infection in brains from NNTC participants with HIV‑associated dementia [50]. Agbey et al [51] have reported expression of HIV *env* RNA in the human CNS, including in astrocytes, in the brains of PWH who had never taken ART.

More recently, similar approaches have been applied to brains from virally suppressed individuals. Ko et al [30] examined postmortem brain tissues from virally suppressed PWH, using DNAscope *in situ* hybridization coupled with cell-type-specific immunostaining to localize HIV DNA that single‑cell resolution. Their central finding was that detectable HIV DNA was confined almost exclusively to CNS myeloid cells, including microglia and perivascular macrophages, with no convincing evidence of HIV DNA within GFAP‑positive astrocytes.

In contrast, Valdebenito et al [52] advanced the concept of astrocytes as CNS HIV reservoirs by demonstrating that astrocytes, although poorly supportive of productive viral replication, can harbor HIV DNA and mediate efficient cell‑to‑cell viral transfer to neighboring cells *in vitro* through gap junctions and other intercellular mechanisms. Much of this work integrated human brain tissues with *in vitro* and *ex vivo* astrocyte models, emphasizing functional consequences of viral presence rather than durable, integrated proviral burden. Compared with Ko *et al*, which focused on the detection of HIV DNA with cellular co-localization,Valdebenito *et al* placed greater weight on low‑level or restricted infection and viral protein/RNA detection, raising the possibility that astrocytes contribute to CNS persistence through non‑canonical or indirect mechanisms rather than acting as a major source of long‑lived, integrated proviruses under suppressive ART.

Cell-type assignment was also addressed in two of the studies from the Churchill group. Cochrane and colleagues [45] performed DNAscope combined with immunofluorescence for the myeloid marker CD68, along with laser-capture microdissection of CD68+ cells, confirming that HIV DNA was present in myeloid cells in brains from virally suppressed donors. Jamal Eddine and colleagues [47] used immunofluorescence detection of HIV p24 (Gag) together with markers for myeloid cells (CD68) and T cells (CD3) to demonstrate HIV Gag protein expression predominantly in myeloid cells in the brains of suppressed individuals. Rare HIV p24-positive T cells were detected, but these were restricted to perivascular or intravascular compartments, consistent with transient trafficking rather than stable parenchymal infection.

Taken together, these studies support a unifying model in which CNS myeloid cells constitute the principal stable HIV reservoir in virally suppressed individuals, while astrocyte involvement is variable and context‑dependent, becoming more apparent in settings of uncontrolled viremia, severe neuroinflammation, or advanced neurocognitive disease. This distinction has important implications for HIV cure strategies, suggesting that eradication efforts must primarily target long‑lived CNS myeloid populations, while also accounting for astrocyte‑mediated viral signaling or protein expression that may sustain neuroinflammation independent of replication‑competent virus.

### Studies on isolated viable brain cells

Given the consistent detection of HIV within cells of the myeloid lineage within the CNS in brain tissue sections, several investigators have isolated myeloid cells (primarily microglia) from PWH to directly assess viral persistence within these long-lived CNS-resident cells. One of the most compelling studies used LG brain tissues to demonstrate that brain-resident microglia can serve as durable reservoirs for replication-competent HIV despite durable systemic plasma viral suppression. Tang and colleagues [35] isolated viable microglia from the parietal cortex of LG donors. They detected both total and integrated HIV DNA, as well as replication-competent virus that could be reactivated *ex vivo*, which was macrophage tropic. These findings provided strong evidence that microglia harbor inducible proviruses even when plasma viremia is undetectable.

Building upon this work, Schlachetzki and colleagues [33] isolated microglia from fresh prefrontal cortical tissue of LG donors and performed single-cell transcriptomic and chromatin accessibility profiling (scRNA-seq and scATAC-seq). HIV RNA and DNA were detected in microglia from 2 of the 3 participants, with an infection frequency of ~0.5% among microglia in the virally suppressed participants, demonstrating the durability of the microglial reservoir at the single-cell level. Complementary findings were reported in a study from five NNTC participants by Nuhn and colleagues [40], including two who were virally suppressed, in which microglia, freshly isolated from the frontal lobe, the subventricular zone, and the occipital lobe regions of brain, were assessed using IPDA. In one suppressed participant, both proviral DNA and intact genomes were detected across all three brain regions examined, whereas in the second suppressed participant, proviral DNA and intact genomes were detected in the frontal and lobes. Another suppressed participant had both proviral DNA and intact genomes only in the subventricular zone. Among the remaining viremic participants, HIV DNA was detected sporadically, and intact proviruses were not observed. Collectively, these studies indicate that microglia can harbor genetically intact, inducible HIV proviruses that persist despite long-term viral suppression, supporting the durability of the CNS reservoir. These findings highlight the challenge posed by CNS-resident myeloid cells, which are long-lived and may be less responsive to systemic ART compared with peripheral immune cell populations.

The possibility of T-cell reservoirs in the CNS remains incompletely defined. Several recent studies demonstrate that HIV-infected CD4⁺ T cells can be detected in the CSF. Single-cell analyses detect HIV RNA+ central memory CD4⁺ T cells in CSF of PWH on ART [53]. Both productive and replication-inactive infection in CSF CD4⁺ T cells occur during acute infection in humans and macaques, but the overall frequency of infected CD4^+^ T cells in the CNS appears low relative to myeloid-lineage cells, and the extent to which these cells constitute a stable reservoir remains uncertain [54]. Technical challenges in isolating rare lymphocyte populations from CSF and brain tissue have limited the number of studies capable of rigorously assessing T-cell infection or persistence in CNS compartments.

### Compartmentalization of HIV sequences in the brain

Studies of HIV env gene sequences from suppressed NNTC participants by Oliveira and colleagues [37] revealed compartmentalization of viral sequences between the brain and paired lymphoid tissues; an earlier study by Rose and colleagues [29] found similar compartmentalization between brain and lymphoid as well as other organ tissues in suppressed NNTC participants. Chaillon and colleagues [31] studied longitudinal blood samples and postmortem tissues from six suppressed LG participants, examining HIV env gene sequences to investigate viral phylogenetics across multiple anatomical compartments. This work revealed persistent viral nucleic acids and evidence of inter-compartmental exchange among lymphoid, gastrointestinal, and CNS tissues. These data support a model of continuous, low-level replication or migration between peripheral and CNS reservoirs that presents additional challenges to HIV cure strategies.

Earlier studies utilizing viral phylogenetics demonstrated that HIV within the CNS can evolve independently from peripheral viral populations, supporting the concept of CNS compartmentalization [29,37]. Phylogenetic analyses have shown that CSF HIV variants often form genetically distinct lineages relative to contemporaneous plasma virus, reflecting localized replication and independent evolutionary trajectories within the CNS. These compartmentalized CSF populations may exhibit unique tropism, most notably macrophage-tropic or highly adapted T-cell–tropic phenotypes, indicating that the CNS can support selective pressures and viral diversification not observed in peripheral blood [55,56]. More recent tissue-based studies extend these findings by providing direct spatial characterization of viral persistence within specific brain regions and CNS cell populations [31,32,37]. These approaches allow for improved localization of HIV reservoirs within microglia, macrophages, and other CNS-associated cells, thereby offering greater resolution than CSF-based analyses alone. Together, these findings strengthen evidence that the CNS functions as a distinct viral reservoir with compartment-specific viral dynamics.

### Summary of brain viral reservoir studies

Collectively, findings from the NNTC, the LG program, and independent cohorts provide evidence demonstrating that HIV persistence in the brain is a consistent and reproducible phenomenon, even among individuals with long-term plasma viral suppression. Across multiple methodological approaches, including ddPCR, IPDA, *in situ* hybridization, near-full-length sequencing, and viable microglia isolation, researchers have repeatedly detected HIV DNA, HIV RNA, and in many cases intact and inducible proviruses within CNS tissues. The consistent detection of viral DNA, RNA, and intact proviruses in virally suppressed individuals indicates that the CNS reservoir is not inert but capable of maintaining and, in some cases, transcriptionally and functionally active competent HIV genomes. Interestingly, other than in brains with HIVE, the level of HIV proviral DNA does not appreciably differ in those participants whose viral load was suppressed [38,41,45]. This parallels observations in the simian immunodeficiency virus (SIV)/nonhuman primate model, where viremia suppression with ART lowered brain SIV RNA levels but not SIV proviral DNA levels [57]. In addition, measures of neuroinflammation in the brains of the treated animals in this study were also decreased. This recognition naturally raises the next critical questions regarding how persistent HIV within the brain contributes to local immune activation and neuroinflammatory processes. The following section focuses on this emerging area of investigation.

Although most CNS HIV reservoir studies focus primarily on viral nucleic acid detection, a limited number have evaluated viral protein expression within brain tissue. Using a multiplex imaging platform, the Eugen’s group demonstrated that HIV-infected cells in the brains of virally suppressed PWH continue to produce detectable viral proteins, including p24, gp120, nef, vpr, and tat, providing direct evidence of persistent translational activity within the CNS reservoir [39]. Jamal Eddine and colleagues demonstrated HIV Gag (p24) expression predominantly within myeloid-lineage cells in brain tissue, supporting persistent viral activity within CNS reservoirs despite ART [47]. Earlier work by Churchill and colleagues reported extensive astrocyte-associated HIV protein detection in individuals with HIV-associated dementia [50]. However, relatively few studies of brains from suppressed PWH have examined HIV-encoded proteins in CNS tissue compared to nucleic acid-based approaches. This discrepancy may partially reflect technical challenges associated with detecting low-abundance viral proteins in postmortem tissue, as well as the greater sensitivity and broader availability of molecular techniques such as PCR-based assays and sequencing approaches [25–28]. Given evidence suggesting viral proteins may contribute to persistent inflammation despite ART, further investigation of HIV protein expression within the CNS remains an important area for future research.

### Neuroinflammation and neurocognitive impairment

Despite effective ART, HIV persistence within the CNS has been repeatedly linked to markers of neuroinflammation and immune activation, several studies have examined this relationship using both bulk and single-cell approaches. Using virally suppressed NNTC donors, Gabuzda *et al* [34] combined IPDA with differential gene-expression and observed that while intact proviral levels did not differ significantly between individuals with and without neurocognitive impairment, brains showing histological evidence of neuroinflammation exhibited a trend toward higher intact-proviral burden. Among the 78 genes examined that are associated with inflammation, stress responses, and white-matter integrity, most were upregulated in brain tissue from PWH compared with that from people without HIV infection, with notable downregulation of genes related to oxidative phosphorylation and oligodendrocyte function.

Using multiplex immunofluorescence, Byrnes and colleagues [38] demonstrated increased expression of interferon-inducible myxovirus resistance protein 1 (Mx-1) and Tumor Necrosis Factor (TNF) in astrocytes and myeloid cells within frontal cortex tissue from both virally suppressed and viremic PWH. Importantly, the frequency of Mx-1–positive myeloid cells correlated with levels of total, intact, and 5′-defective HIV proviral DNA in virally-suppressed participants, supporting a role for persistent proviral burden with local immune activation of HIV in the CNS.

In a study of participants in the LG cohort who had long-term viral suppression, Trufino and colleagues [58] found that despite consistent detection of HIV DNA and RNA across all CNS regions, quantitative measures of reservoir size and transcriptional activity showed no association with cognitive performance. Interestingly, while CNS HIV burden alone did not account for neurocognitive variability, their examination of the T-cell receptor repertoire in different brain regions revealed linkages to specific cognitive function, suggesting neuroimmune factors may drive cognitive deficits in suppressed PWH.

Single-cell omics approaches have extended these observations on neuroinflammation. In the study of Schlachetzki and colleagues [33], the HIV RNA-positive microglia were enriched for interferon-stimulated and pro-inflammatory gene signatures, demonstrating that infected microglia remain transcriptionally active under suppressive ART. Building on these insights, Tang and colleagues [59] first demonstrated of increased type I interferon signaling in neurocognitively impaired NNTC participants (on ART but not all suppressed) using proteomics. Next, they used scRNA-seq to examine two virally-suppressed participants (one from the LG, the other through the National Disease Research Interchange following LG protocols) and found transcriptome evidence of persistent type I interferon signaling in the cells, providing mechanistic evidence that sustained interferon activity may bridge latent infection and neuroinflammation [59].

A recent study by Wilson and colleagues [60] examined the combined effects of HIV infection and substance use disorder (SUD) on neuronal and microglial gene transcriptional programs in the ventral midbrain using tissue from 90 NNTC participants. The cohort included 28 participants without HIV, 30 virally suppressed PWH, and 32 viremic PWH, with each group further stratified by the presence of opioid or cocaine SUD. Virally suppressed PWH with SUD were found to have a dysregulated transcriptional profile in dopaminergic neurons, and an altered microglial transcriptome consistent with increased susceptibility to HIV infection, although detection of HIV was not reported.

Together, these studies highlight a converging theme: even with effective systemic viral suppression, the CNS remains an immunologically distinct environment in which infected microglia and macrophages can sustain low-level chronic inflammation. Persistent interferon signaling, cellular activation, and ongoing proviral transcription all point to neuroinflammation as both a marker and a potential driver of CNS reservoir maintenance. These processes may help explain the cognitive deficits observed in some PWH, providing the foundation for the next section’s focus on neurocognitive outcomes.

### Integrated evidence for HIV persistence in the brain

In the modern ART era, neuropathology in PWH, including those with viral suppression, is generally limited to mild, nonspecific findings, such as low-grade gliosis, microglial activation, and sparse perivascular macrophages, in contrast to the severe HIVE, multinucleated giant cells, and extensive white-matter damage that characterized the pre-ART period [34,61,62]. This broader pathological context underscores that the viral signals detected in brain tissue today arise within a landscape of attenuated but not absent neuroinflammation, highlighting the importance of understanding persistent tissue-resident reservoirs rather than the dramatic encephalitic pathology that dominated the pre-ARTera.

These autopsy-based studies reveal that HIV DNA and RNA remain detectable in the brains of virally suppressed PWH. As summarized in S5A Table, brain tissue from 38 of the virally suppressed NNTC participants has been assayed in one or more studies focused on proviral DNA load, and 32 (84%) had at least one brain region in which HIV DNA was detected. The median level of HIV proviral DNA was 2.53 copies per 10^6^ cells. IPDA-based analyses (S5B Table) have been performed on 30 virally suppressed NNTC participants, with HIV DNA detectable in all 30 (100%) samples, with a median of 3.55% being intact. When examining specimens from suppressed NNTC participants in either set of techniques, samples from 43 of the 45 (96%) had HIV proviral DNA detected.

While there are indications that the distribution of HIV DNA varies by region, the limited number of studies assessing multiple regions within the same individuals using uniform methodologies precludes definitive conclusions regarding regional specificity. The field lacks large-scale, region-by-region analyses conducted across a sufficiently large number of participants using consistent assay platforms. As such, it remains unclear whether differences across brain regions reflect true regional specificity of infection or heterogeneous distribution that varies between individuals. Despite potential region-specific patterns, it is important to underscore that the absolute burden of infected cells is exceedingly low, with most assays detecting only rare events near the limits of quantification, such that much of the field necessarily relies on binary detection rather than robust quantitative distributions, reflecting the profound degree of viral suppression achieved in the CNS on long-term ART.

Sequence analyses reveal various patterns of viral compartmentalization in PWH [36,63]. In some individuals, brain-derived env sequences form distinct clusters, suggesting localized evolution within CNS tissues [63]. In others, viral variants are widely distributed across the brain, lymphoid, gastrointestinal, and peripheral tissues, indicating a lack of strict compartmental separation [36]. Studies from both the NNTC and LG cohorts demonstrate that viral genomes can persist, evolve, and traffic between migrate among anatomical compartments even under long-term ART, potentially through infected cell migration, clonal expansion, or low-level replication. These observations blur traditional distinctions between “brain” and “systemic” reservoirs.

## Discussion

The NNTC and LG studies have played a central role in advancing the understanding of HIV persistence in the CNS. These resources have enabled investigators to move beyond blood-based analyses and directly interrogate viral persistence within CNS sanctuary sites using increasingly sophisticated molecular and cellular approaches.

The NNTC provides long-term clinical and neuropathological context, allowing viral and molecular findings to be linked to cognitive trajectories and comorbidities over time, and the availability to requestors of brain, other tissues, and biofluids from well-characterized participants. Fixed and frozen brain tissues have been used for a variety of techniques, including snRNA-seq. Complementing the NNTC, the LG program, a rapid-autopsy cohort designed to investigate HIV persistence at the end of life, has provided unique insights into CNS reservoirs through near-death sampling of individuals maintained on suppressive ART until death. The short postmortem intervals achieved in this cohort preserve molecular integrity and cellular viability, enabling functional assays, single-cell analyses, and direct assessment of viral inducibility across multiple tissues, including the brain. Overall, findings from the NNTC and LG cohorts converge to demonstrate that intact proviral genomes and viral RNA persist in the brains of virally suppressed individuals, as shown by ddPCR, IPDA, near-full-length sequencing, and single-cell approaches. Together, these studies bridge clinical observation and mechanistic biology, providing a more complete understanding of how HIV persists in the brain despite effective systemic therapy.

Despite major advances enabled by autopsy-based studies, neuroHIV research remains fundamentally constrained by the limited availability of brain tissue compared with more extensively studied neurodegenerative diseases. In Alzheimer’s disease (AD), large, coordinated brain banking efforts and standardized neuropathologic assessment frameworks have enabled the accumulation of thousands of well-characterized cases, facilitating statistically powered analyses, regional comparisons, and cross-cohort validation [64]. In contrast, studies of HIV-associated brain pathology have relied on far smaller numbers of cases, reflecting both historical declines in HIV-related mortality in the ART era and persistent barriers to autopsy enrollment and tissue collection in PWH, as well as the relative increased prevalence of aged individuals with disorders such as AD compared to those with HIV [65,66]. Resources such as the NNTC and LG have helped mitigate these challenges. However, the overall scale of available neuroHIV brain repositories remains modest relative to those supporting AD research [66]. This disparity necessarily limits statistical power, increases reliance on binary detection outcomes, and complicates comparisons across studies that may analyze overlapping or partially overlapping tissue sets. These constraints must be considered when interpreting reported estimates of viral persistence, regional distribution, or associations with neuropathology and neuroinflammation, and they underscore the importance of expanding and sustaining dedicated neuroHIV brain banking efforts to more clearly define the role of the CNS as an HIV reservoir in the ART era.

While this review focuses on evidence of HIV persistence within the CNS, it is important to emphasize that most autopsy-based studies discussed here are designed to detect and localize viral nucleic acids or proteins, rather than to define the molecular mechanisms responsible for reservoir establishment or maintenance. As such, these studies should be interpreted primarily as identifying the presence, cellular distribution, and anatomical context of CNS reservoirs under ART, rather than demonstrating causative pathways. Nevertheless, when considered collectively, these observations generate testable mechanistic hypotheses. For example, the predominance of myeloid-lineage cells among HIV-positive CNS cells, the low-level or restricted infection profiles reported in astrocytes, and evidence for cell-to-cell viral transfer suggest that viral persistence in the brain may be supported by non-canonical infection states, indirect propagation, or prolonged survival of long-lived resident cells rather than ongoing productive replication. Similarly, the restrictive nature of the blood–brain barrier with respect to immune surveillance and penetration of some antiretrovirals may permit persistent low-level transcription or protein expression without overt systemic viral rebound. These inferences do not establish mechanisms but instead delineate conceptual frameworks that can be rigorously tested using experimental model systems. Explicitly distinguishing reservoir detection from mechanistic validation is essential for appropriately interpreting CNS autopsy data and for guiding future studies aimed at defining how CNS-specific cellular and anatomical features shape HIV latency and persistence in the ART era.

## Perspectives, limitations, and future directions

Despite the transformative effect of ART, the brain remains one of the most complex HIV reservoirs. CSF sampling provides only a partial window into the CNS and cannot capture the regional and cellular diversity revealed by autopsy studies. Work from the NNTC, LG, and independent cohorts shows that HIV DNA, HIV RNA, and intact proviruses persist in brain tissues of virally suppressed individuals, and that microglia can produce macrophage-tropic replication-competent virus *ex vivo*. These findings underscore the need for therapeutic strategies that effectively penetrate and act within the CNS.

Key uncertainties remain and are summarized in Table 3. The extent to which astrocytes are productively infected remains debated; while some studies detect HIV DNA or protein in astrocytes, definitive evidence of productive infection is limited. The blood–brain barrier restricts drug penetration and immune cell trafficking, potentially allowing low-level transcription to persist undetected. In addition, reservoir cell types differ by compartment: myeloid-lineage cells dominate in the CNS, whereas T cells dominate in lymphoid tissues, suggesting distinct latency mechanisms and immune environments. HIV persistence in the CNS is reinforced by latency mechanisms that restrict viral transcription through chromatin condensation, transcription-factor sequestration, and integration into repressive genomic domains. Recent single-nucleus studies further demonstrate that infected microglia exhibit 3D-genome remodeling and activation-linked epigenetic states that may -stabilize- latent proviruses while permitting intermittent reactivation [67].

**Table 3:** Gaps and issues. Gaps in our knowledge on the brain as a reservoir and issues regarding the ability of brain banks to meet investigators’ needs.

Domain	Unresolved Gap	Implications for HIV Cure Research
**Cellular Identity of CNS Reservoirs**	It remains uncertain which brain cell types harbor intact, replication-competent proviruses versus defective genomes, particularly under long-term ART	Limits identification of true, actionable CNS reservoirs and hinders cell-type–specific cure strategies
	While microglia are recognized as the dominant reservoir, the contribution of perivascular macrophages, brain-infiltrating T cells, and other potential CNS cell types to rebound viremia and ongoing injury is poorly defined	Different cell types vary in lifespan, trafficking, immune surveillance, and drug sensitivity
**Reservoir Dynamics Across the Disease Course**	Very limited data on how CNS reservoir size, clonality, and transcriptional activity change across acute infection, early ART initiation, and decades of viral suppression	Prevents modeling of reservoir establishment, maintenance, and long-term evolution in the brain
	Unclear how frequently CNS reservoirs reactivate during suppressive ART	Limits understanding of ongoing neuroinflammation and subclinical injury
**Contribution to Systemic Rebound**	It remains unclear how often CNS reservoirs contribute to systemic viral rebound after treatment interruption	Critical gap for determining whether CNS reservoirs must be directly targeted for durable remission
**Impact of Cure Strategies on the CNS**	Most HIV cure trials pay limited attention to CNS reservoirs	Risks overestimating efficacy of cure interventions based solely on peripheral measurements
	The effects of latency-reversing or latency-silencing strategies on the brain reservoir are largely unknown	Potential for unrecognized CNS toxicity or failure to impact a key reservoir
	There is little preclinical work testing whether CNS-directed interventions can safely modulate brain reservoirs	Slows translation of CNS-safe cure approaches
**Brain Bank Representation and Bias**	Brain banks are biased toward advanced disease (HIV-related or other causes) and individuals who consent to autopsy	Limits generalizability to virally suppressed PWH living long-term with ART
**Tissue Quality and Heterogeneity**	Postmortem interval, agonal factors, and variable fixation/freezing protocols introduce substantial heterogeneity	Complicates cross-study comparisons and interpretation of negative or low-signal findings
**Standardization of Tissue Processing**	Lack of standardized SOPs for region-specific processing (e.g., small blocks, OCT vs dry-snap freezing, short-duration fixation for FFPE, cryopreservation)	Reduces compatibility with modern multi-omic and spatial approaches
	Limited parallel tissue collection optimized for advanced techniques (e.g., spatial transcriptomics, spatial proteomics)	Constrains discovery of spatial and microenvironmental reservoir biology
**Reservoir Measurement Infrastructure**	Absence of embedded HIV reservoir measurement pipelines within brain banks	Forces investigators to independently perform basic annotation, increasing cost and variability
	Lack of standardized protocols for quantifying HIV DNA, RNA, and intact proviruses	Prevents harmonized data reporting and cross-cohort synthesis
**Quality Assurance and Control**	Standardized QA/QC metrics are often absent or inconsistently applied	Limits confidence in data quality and reproducibility
**Cross-Scale Integration**	Limited linkage between postmortem molecular findings and antemortem neuroimaging (MRI, PET)	Restricts mechanistic connections between reservoirs, neuroinflammation, and clinical outcomes
**Cell Availability**	Availability of fresh and cryopreserved viable CNS cell types is limited	Severely constrains functional, inducibility, and therapeutic testing
**Sustainability of Brain Banks**	High fixed costs of acquisition, processing, and storage combined with unpredictable demand	Threatens long-term viability of brain banks essential for CNS reservoir research
**Relationship between HIV reservoirs and neuropathology**	Existing datasets from NNTC and LG contain extensive virologic and clinical information, but integrated analyses linking reservoir measurements with neuropathology,neuroimaging, and neurocognitive outcomes remain limited.	Important for contextualizing the broader significance of virologic findings in the brain.

Methodological challenges also complicate interpretation. Post-mortem intervals, tissue quality, level of intactness of nucleic acids, and assay sensitivity vary across studies, as do palliative care, agonal state, and other conditions prior to death. Current approaches, such as ddPCR and IPDA, cannot definitively measure replication competence. Few datasets link molecular signatures to longitudinal clinical or neuroimaging outcomes. Advances in single-nucleus multi-omics, RNAscope, immunohistochemistry/immunofluorescence, spatial transcriptomics, and spatial proteomics will be essential to map viral and host interactions at higher resolution. Rapid-autopsy programs with short postmortem intervals and access to fresh tissues, such as LG, are uniquely positioned to support such emerging technologies in addition to viable-cell assays that together may help define the cellular and anatomical basis of HIV persistence in the CNS.

Autopsy-based cohorts are enriched for individuals with advanced disease and significant comorbidities. These factors may have a disproportionate impact on measures of neuroinflammation and glial activation, which are highly sensitive to systemic illness, terminal events, and treatment-associated immune perturbations [29,39]. In contrast, available evidence suggests that the presence and anatomical distribution of CNS HIV DNA and RNA, particularly when assessed using robust bulk or localization-based assays are less directly influenced by these comorbid conditions and instead reflect long-term reservoir establishment within resident CNS cells [31,32]. Accordingly, associations between CNS HIV reservoirs and inflammatory or neuropathologic readouts should be interpreted with caution, as comorbidities may amplify or obscure inflammatory signals without necessarily altering reservoir size or persistence. This distinction underscores the importance of separating reservoir detection from downstream host response measures when interpreting autopsy-based CNS studies.

End-of-life conditions constitute a critical contextual factor when interpreting autopsy-based HIV measurements, as NNTC and LG participants encompass individuals with terminal neurodegenerative disease, hematologic malignancy, systemic inflammation, and other conditions that may differentially influence tissue-resident HIV reservoirs and host immune activation. Variability in time for ART cessation, postmortem interval, agonal state, terminal medical complications, and palliative care interventions such as comfort and pain relief medications as well as stopping ART, can further affect tissue integrity, RNA preservation, neuroimmune signaling, and potentially viral recrudescence, thereby complicating the interpretation of cell-associated HIV RNA, inflammatory biomarkers, transcriptional activity, and neuropathological findings. Prolonged postmortem interval contributes to RNA degradation, while terminal systemic inflammation, sepsis, organ failure, and medications such as sedatives or opioids may modulate immune responses in ways that are difficult to disentangle from underlying virologic processes. These inherent limitations of autopsy-based research should be carefully considered when comparing tissue measurements across participants or cohorts, particularly given that such samples may not fully reflect patterns observed in younger, healthier, or earlier-treated PWH (S4 Table).

Brain-bank–derived cohorts inherently over-represent individuals with more advanced disease and those who ultimately consent to autopsy, which limits generalizability to the broader population of long-term treated PWH, particularly younger, healthier, and earlier-treated individuals. Although the NNTC originated as a brain-bank network with participant characterization, it has long functioned as a prospective long-term cohort with continued longitudinal systematic clinical, neurocognitive, and laboratory characterization as ART outcomes improved, providing a more representative clinical context than traditional autopsy-only collections. Even with this longitudinal design, the core autopsy component introduces sampling bias that must be considered when interpreting associations between brain reservoirs**,** neuroinflammation**,** and neurocognitive outcomes**,** which may differ from patterns observed in younger or less comorbid populations receiving contemporary ART.

Large collaborative efforts, such as the Single Cell Opioid Responses in the Context of HIV (SCORCH) consortium, which examines the impact of substance abuse and HIV infection, separately and together [68], have made great use of the NNTC. Exemplifying the scale and integration required to advance the field, the NNTC has to date shipped over 1,400 autopsy brain specimens representing multiple brain regions from 350 NNTC participants to this consortium. SCORCH work includes the recent publication discussed above on the impact of substance use and HIV on the ventral midbrain [60], highlighting the importance of incorporating comorbidities into models of CNS HIV persistence. Standardized processed data from these efforts will be publicly available through the NEMO data portal (https://scorch-portal.nemoarchive.org), facilitating broader discovery and replication. The raw SCORCH datasets are deposited in the NIH Database of Genotypes and Phenotypes (dbGaP), which provides controlled-access archiving of the potentially identifying raw genomic data with secure distribution of human genomic data to qualified investigators.

Regional differences in HIV detection likely reflect both limited sampling and the underlying neuroanatomical and cellular heterogeneity that has been recognized since the earliest pre-ART neuropathologic descriptions of HIV-associated CNS injury [61,69,70]. ART-era autopsy studies demonstrating heterogeneous, region-specific detection of HIV DNA and RNA in brain tissue are broadly consistent with these [46,71]. However, the degree of regional variability observed in contemporary cohorts is substantially less dramatic than would have been predicted from pre-ART neuropathology, likely reflecting the combined effects of suppressive ART, low overall abundance of infected CNS cells, sampling limitations, and methodological variability intrinsic to autopsy-based studies. Overall, anatomical context remains biologically plausible as a determinant of HIV persistence and transcriptional activity within the CNS, but rigorous region-matched, single-cell, multi-omic, and spatial analyses will define how microglial ontogeny, metabolic gradients, vascular niches, and local immune states shape the CNS reservoir during long-term ART.

Finally, the extent to which CNS reservoirs contribute to systemic viral rebound following ART interruption remains uncertain. Although CNS-derived sequences have occasionally been detected in rebound plasma, their relative contribution compared with lymphoid and other tissue reservoirs is unclear. Resolving this question will require coordinated analytic treatment interruption studies that incorporate CNS sampling, viral phylogenetics, and longitudinal clinical and immunologic assessments. Integrating molecular findings with functional and neuropathological data from cohorts such as the NNTC and LG will be critical to defining the role of the brain reservoir in both systemic persistence and neurocognitive outcomes.

## Conclusion

Postmortem studies from the NNTC, LG, and related cohorts provide definitive evidence that the brain remains a persistent HIV reservoir despite long‑term viral suppression. The presence of intact proviruses, infected microglia, ongoing transcriptional activity, and chronic neuroinflammation highlights the CNS as a critical, biologically active site of HIV persistence. Future HIV cure strategies must explicitly target this compartment, requiring deeper mechanistic insight and integrated clinical–molecular research.

## Supporting information

S1 Table Baseline and longitudinal assessments and biobanking in NNTC participants. (XLSX)

S2 Table Autopsy neuropathology, organ pathology, and biobankinginNNTC decedents. (XLSX)

S3 Table Criteria used for the virally-suppressed autopsy cohorts at the NNTC. The groups are not mutually exclusive, as 33 donors meet both criteria.
(XLSX)

S4 Table Donors in the NNTC virallysuppressed cohorts. Donors are listed by pseudoGUID, with viral, neurocognitive, and neuropathological information. For last plasma viral load: LOD, lower limit of detection. For last neurocognitive status: NL, normal/not impaired; HAD, HIV-associated dementia; MND, mild neurocognitive disorder; YEARS, asymptomatic neurocognitive impairment; NPI-THE-neuropsychological impairment with other non-HIV etiology. For comorbidities: 1-Hypertension, 2-Diabetes, 3-Hyperlipidemia, 4-Viral Hepatitis, 5-End Stage Liver Disease, 6-Chronic Renal Disease, 7-Cardiac Disease, 8-Chronic Obstructive Pulmonary Disease, 9-Cerebrovascular Disease, 10-Non-AIDS Defining Cancer, 11-Lipodystrophy, 12-Tobacco Smoking. ^indicates missing information on 12. *indicates information on most other comorbidities missing. For suppressed criteria met: 1 and 2 indicate the criteria in S3 Table.
(XLSX)

S5 Table (A) Brain proviral DNA load and **(B)** Brain IPDAinNNTC suppressed cohorts. Shading of participant pseudoGUID indicates the cases where data are available. ND, not detected.
(XLSX)

## References

[1] Chun TW, Stuyver L, Mizell SB, Ehler LA, Mican JA, Baseler M, et al. Presence of an inducible HIV-1 latent reservoir during highly active antiretroviral therapy. Proc Natl Acad Sci U S A. 1997;94(24):13193–7. doi: 10.1073/pnas.94.24.13193 9371822 PMC24285

[2] Siliciano RF, Greene WC. HIV latency. Cold Spring Harb Perspect Med. 2011;1(1):a007096. doi: 10.1101/cshperspect.a007096 22229121 PMC3234450

[3] Estes JD, Kityo C, Ssali F, Swainson L, Makamdop KN, Del Prete GQ, et al. Defining total-body AIDS-virus burden with implications for curative strategies. Nat Med. 2017;23(11):1271–6. doi: 10.1038/nm.4411 28967921 PMC5831193

[4] Li JZ, Etemad B, Ahmed H, Aga E, Bosch RJ, Mellors JW, et al. The size of the expressed HIV reservoir predicts timing of viral rebound after treatment interruption. AIDS. 2016;30(3):343–53. doi: 10.1097/QAD.0000000000000953 26588174 PMC4840470

[5] Hatano H. Immune activation and HIV persistence: considerations for novel therapeutic interventions. Curr Opin HIV AIDS. 2013;8(3):211–6. doi: 10.1097/COH.0b013e32835f9788 23454864 PMC4041488

[6] Deeks SG, Lewin SR, Havlir DV. The end of AIDS: HIV infection as a chronic disease. Lancet. 2013;382(9903):1525–33. doi: 10.1016/S0140-6736(13)61809-7 24152939 PMC4058441

[7] Deeks SG. HIV infection, inflammation, immunosenescence, and aging. Annu Rev Med. 2011;62:141–55. doi: 10.1146/annurev-med-042909-093756 21090961 PMC3759035

[8] Guaraldi G, Orlando G, Zona S, Menozzi M, Carli F, Garlassi E, et al. Premature age-related comorbidities among HIV-infected persons compared with the general population. Clin Infect Dis. 2011;53(11):1120–6. doi: 10.1093/cid/cir627 21998278

[9] Ho DD, Rota TR, Schooley RT, Kaplan JC, Allan JD, Groopman JE, et al. Isolation of HTLV-III from cerebrospinal fluid and neural tissues of patients with neurologic syndromes related to the acquired immunodeficiency syndrome. N Engl J Med. 1985;313(24):1493–7. doi: 10.1056/NEJM198512123132401 2999591

[10] Navia BA, Jordan BD, Price RW. The AIDS dementia complex: I. Clinical features. Ann Neurol. 1986;19(6):517–24. doi: 10.1002/ana.410190602 3729308

[11] Williams DW, Eugenin EA, Calderon TM, Berman JW. Monocyte maturation, HIV susceptibility, and transmigration across the blood brain barrier are critical in HIV neuropathogenesis. J Leukoc Biol. 2012;91(3):401–15. doi: 10.1189/jlb.0811394 22227964 PMC3289493

[12] Arrildt KT, LaBranche CC, Joseph SB, Dukhovlinova EN, Graham WD, Ping L-H, et al. Phenotypic correlates of HIV-1 macrophage tropism. J Virol. 2015;89(22):11294–311. doi: 10.1128/JVI.00946-15 26339058 PMC4645658

[13] Letendre SL, Ellis RJ, Ances BM, McCutchan JA. Neurologic complications of HIV disease and their treatment. Top HIV Med. 2010;18(2):45–55. 20516524 PMC5077300

[14] Robert SM, Reeves BC, Kiziltug E, Duy PQ, Karimy JK, Mansuri MS, et al. The choroid plexus links innate immunity to CSF dysregulation in hydrocephalus. Cell. 2023;186(4):764-785.e21. doi: 10.1016/j.cell.2023.01.017 36803604 PMC10069664

[15] Antinori A, Arendt G, Becker JT, Brew BJ, Byrd DA, Cherner M, et al. Updated research nosology for HIV-associated neurocognitive disorders. Neurology. 2007;69(18):1789–99. doi: 10.1212/01.WNL.0000287431.88658.8b 17914061 PMC4472366

[16] Nightingale S, Ances B, Cinque P, Dravid A, Dreyer AJ, Gisslén M, et al. Cognitive impairment in people living with HIV: consensus recommendations for a new approach. Nat Rev Neurol. 2023;19(7):424–33. doi: 10.1038/s41582-023-00813-2 37311873

[17] Ellis RJ, Calero P, Stockin MD. HIV infection and the central nervous system: a primer. Neuropsychol Rev. 2009;19(2):144–51. doi: 10.1007/s11065-009-9094-1 19415500 PMC2690832

[18] Saylor D, Dickens AM, Sacktor N, Haughey N, Slusher B, Pletnikov M, et al. HIV-associated neurocognitive disorder--pathogenesis and prospects for treatment. Nat Rev Neurol. 2016;12(4):234–48. doi: 10.1038/nrneurol.2016.27 26965674 PMC4937456

[19] Heithoff AJ, Totusek SA, Le D, Barwick L, Gensler G, Franklin DR. The integrated National NeuroAIDS Tissue Consortium database: a rich platform for neuroHIV research. Database. 2019;2019. 10.1093/database/bay134 PMC6323298 30624650

[20] Perry KE, Taylor J, Patel H, Javadi SS, Mathur K, Kaytes A, et al. “[It] is now my responsibility to fulfill that wish:” Clinical and rapid autopsy staff members’ experiences and perceptions of HIV reservoir research at the end of life. PLoS One. 2020;15(11):e0242420. doi: 10.1371/journal.pone.0242420 33206710 PMC7673534

[21] Ahmed A, Taylor J, Tran W, Swaitch S, Ndukwe SO, Lau R, et al. Motivations, acceptability and ethical considerations for interventional HIV cure research at the end of life: perspectives from long-term survivors of HIV in the United States. BMC Med Ethics. 2025;26(1):112. doi: 10.1186/s12910-025-01253-x 40849625 PMC12374411

[22] Ndukwe SO, Patel H, Shelton B, Concha-Garcia S, Dullano C, Solso S, et al. People with HIV at the end-of-life and their next-of-kin/loved ones are willing to participate in interventional HIV cure-related research. AIDS. 2024;38(2):235–43. doi: 10.1097/QAD.0000000000003754 37861674 PMC10842373

[23] Dubé K, Shelton B, Patel H, Ndukwe SO, Concha-Garcia S, Dullano C, et al. Perceived risks and benefits of enrolling people with HIV at the end of life in cure research in Southern California, United States. J Virus Erad. 2023;9(2):100328. doi: 10.1016/j.jve.2023.100328 37440872 PMC10334343

[24] Prakash K, Gianella S, Dubé K, Taylor J, Lee G, Smith DM. Willingness to participate in HIV research at the end of life (EOL). PLoS One. 2018;13(7):e0199670. doi: 10.1371/journal.pone.0199670 30036365 PMC6056048

[25] Moar P, Premeaux TA, Atkins A, Elephant LC. The latent HIV reservoir: current advances in genetic sequencing approaches. mBio 2023; 14 (5): e0134423. doi: 10.1128/mbio.01344-23 37811964 PMC10653892

[26] Mbhele N, Chimukangara B, Tyers L, Maldarelli F, Redd AD. Advancements in single-cell techniques for examining the HIV reservoir: pathways to a cure. mBio. 2025;16(7):e0065525. doi: 10.1128/mbio.00655-25 40488537 PMC12239569

[27] Abdel-Mohsen M, Richman D, Siliciano RF, Nussenzweig MC, Howell BJ, Martinez-Picado J, et al. Recommendations for measuring HIV reservoir size in cure-directed clinical trials. Nat Med. 2020;26(9):1339–50. doi: 10.1038/s41591-020-1022-1 32895573 PMC7703694

[28] Sanders-Beer BE, Archin NM, Brumme ZL, Busch MP, Deleage C, O’Doherty U, et al. Current HIV/SIV reservoir assays for preclinical and clinical applications: recommendations from the experts 2022 NIAID workshop summary. AIDS Res Hum Retroviruses. 2023;:10.1089/AID.2022.0188. doi: 10.1089/AID.2022.0188 37126090

[29] Rose R, Lamers SL, Nolan DJ, Maidji E, Faria NR, Pybus OG, et al. HIV Maintains an evolving and dispersed population in multiple tissues during suppressive combined antiretroviral therapy in individuals with cancer. J Virol. 2016;90(20):8984–93. doi: 10.1128/JVI.00684-16 27466425 PMC5044847

[30] Ko A, Kang G, Hattler JB, Galadima HI, Zhang J, Li Q, et al. Macrophages but not astrocytes harbor HIV DNA in the brains of HIV-1-infected aviremic individuals on suppressive antiretroviral therapy. J Neuroimmune Pharmacol. 2019;14(1):110–9. doi: 10.1007/s11481-018-9809-2 30194646 PMC6391194

[31] Chaillon A, Gianella S, Dellicour S, Rawlings SA, Schlub TE, De Oliveira MF, et al. HIV persists throughout deep tissues with repopulation from multiple anatomical sources. J Clin Invest. 2020;130(4):1699–712. doi: 10.1172/JCI134815 31910162 PMC7108926

[32] Sun W, Rassadkina Y, Gao C, Collens SI, Lian X, Solomon IH, et al. Persistence of intact HIV-1 proviruses in the brain during antiretroviral therapy. Elife. 2023;12:RP89837. doi: 10.7554/eLife.89837 37938115 PMC10631759

[33] Schlachetzki JC, Gianella S, Ouyang Z, Lana AJ, Yang X, O’Brien S, et al. Gene expression and chromatin conformation of microglia in virally suppressed people with HIV. Life Sci Alliance. 2024;7(10):e202402736. doi: 10.26508/lsa.202402736 39060113 PMC11282357

[34] Gabuzda D, Yin J, Misra V, Chettimada S, Gelman BB. Intact proviral DNA analysis of the brain viral reservoir and relationship to neuroinflammation in people with HIV on suppressive antiretroviral therapy. Viruses. 2023;15(4):1009. doi: 10.3390/v15041009 37112989 PMC10142371

[35] Tang Y, Chaillon A, Gianella S, Wong LM, Li D, Simermeyer TL, et al. Brain microglia serve as a persistent HIV reservoir despite durable antiretroviral therapy. J Clin Invest. 2023;133(12):e167417. doi: 10.1172/JCI167417 37317962 PMC10266791

[36] Brese RL, Gonzalez-Perez MP, Koch M, O’Connell O, Luzuriaga K, Somasundaran M, et al. Ultradeep single-molecule real-time sequencing of HIV envelope reveals complete compartmentalization of highly macrophage-tropic R5 proviral variants in brain and CXCR4-using variants in immune and peripheral tissues. J Neurovirol. 2018;24(4):439–53. doi: 10.1007/s13365-018-0633-5 29687407 PMC7281851

[37] Oliveira MF, Pankow A, Vollbrecht T, Kumar NM, Cabalero G, Ignacio C, et al. Evaluation of archival HIV DNA in brain and lymphoid tissues. J Virol. 2023; 97 (6): e0054323. doi: 10.1128/jvi.00543-23 37184401 PMC10308944

[38] Byrnes SJ, Jamal Eddine J, Zhou J, Chalmers E, Wanicek E, Osman N, et al. Neuroinflammation associated with proviral DNA persists in the brain of virally suppressed people with HIV. Front Immunol. 2025;16:1570692. doi: 10.3389/fimmu.2025.1570692 40469287 PMC12135078

[39] Donoso M, D’Amico D, Valdebenito S, Hernandez CA, Prideaux B, Eugenin EA. Identification, quantification, and characterization of HIV-1 reservoirs in the human brain. Cells. 2022;11(15). 10.3390/cells11152379 PMC9367788 35954221

[40] Nühn MM, Gumbs SBH, Schipper PJ, Drosou I, Gharu L, Buchholtz NVEJ, et al. Microglia exhibit a unique intact HIV reservoir in human postmortem brain tissue. Viruses. 2025;17(4):467. doi: 10.3390/v17040467 40284910 PMC12030925

[41] Mohammadzadeh N, Roda W, Branton WG, Clain J, Rabezanahary H, Zghidi-Abouzid O, et al. Lentiviral infections persist in brain despite effective antiretroviral therapy and neuroimmune activation. mBio. 2021;12(6):e0278421. doi: 10.1128/mBio.02784-21 34903055 PMC8669467

[42] Chung HK, Hattler JB, Narola J, Babbar H, Cai Y, Abdel-Mohsen M, et al. Development of droplet digital pcr-based assays to quantify hiv proviral and integrated DNA in brain tissues from viremic individuals with encephalitis and virally suppressed aviremic individuals. Microbiol Spectr. 2022;10(1):e0085321. doi: 10.1128/spectrum.00853-21 35019681 PMC8754137

[43] Bruner KM, Wang Z, Simonetti FR, Bender AM, Kwon KJ, Sengupta S, et al. A quantitative approach for measuring the reservoir of latent HIV-1 proviruses. Nature. 2019;566(7742):120–5. doi: 10.1038/s41586-019-0898-8 30700913 PMC6447073

[44] Reeves DB, Gaebler C, Oliveira TY, Peluso MJ, Schiffer JT, Cohn LB, et al. Impact of misclassified defective proviruses on HIV reservoir measurements. Nat Commun. 2023;14(1):4186. doi: 10.1038/s41467-023-39837-z 37443365 PMC10345136

[45] Cochrane CR, Angelovich TA, Byrnes SJ, Waring E, Guanizo AC, Trollope GS, et al. Intact HIV proviruses persist in the brain despite viral suppression with ART. Ann Neurol. 2022;92(4):532–44. doi: 10.1002/ana.26456 35867351 PMC9489665

[46] Angelovich TA, Cochrane CR, Zhou J, Tumpach C, Byrnes SJ, Jamal Eddine J, et al. Regional analysis of intact and defective HIV proviruses in the brain of viremic and virally suppressed people with HIV. Ann Neurol. 2023;94(4):798–802. doi: 10.1002/ana.26750 37493435 PMC10914117

[47] Jamal Eddine J, Angelovich TA, Zhou J, Byrnes SJ, Tumpach C, Saraya N, et al. HIV transcription persists in the brain of virally suppressed people with HIV. PLoS Pathog. 2024;20(8):e1012446. doi: 10.1371/journal.ppat.1012446 39116185 PMC11335163

[48] Wiley CA, Schrier RD, Nelson JA, Lampert PW, Oldstone MB. Cellular localization of human immunodeficiency virus infection within the brains of acquired immune deficiency syndrome patients. Proc Natl Acad Sci U S A. 1986;83(18):7089–93. doi: 10.1073/pnas.83.18.7089 3018755 PMC386658

[49] Gabuzda DH, Ho DD, de la Monte SM, Hirsch MS, Rota TR, Sobel RA. Immunohistochemical identification of HTLV-III antigen in brains of patients with AIDS. Ann Neurol. 1986;20(3):289–95. doi: 10.1002/ana.410200304 3532930

[50] Churchill MJ, Wesselingh SL, Cowley D, Pardo CA, McArthur JC, Brew BJ, et al. Extensive astrocyte infection is prominent in human immunodeficiency virus-associated dementia. Ann Neurol. 2009;66(2):253–8. doi: 10.1002/ana.21697 19743454

[51] Agbey C, Walton S, Campbell LA, Nath A, Smith B, Snow J, et al. Expression of HIV envelope protein in the human central nervous system. AIDS. 2025;39(7):788–97. doi: 10.1097/QAD.0000000000004150 39932701 PMC12064364

[52] Valdebenito S, Castellano P, Ajasin D, Eugenin EA. Astrocytes are HIV reservoirs in the brain: a cell type with poor HIV infectivity and replication but efficient cell-to-cell viral transfer. J Neurochem. 2021;158(2):429–43. doi: 10.1111/jnc.15336 33655498 PMC11102126

[53] Wang M, Yoon J, Reisert H, Das B, Orlinick B, Chiarella J, et al. HIV-1-infected T cell clones are shared across cerebrospinal fluid and blood during ART. JCI Insight. 2024;9(7):e176208. doi: 10.1172/jci.insight.176208 38587074 PMC11128194

[54] Sharma V, Creegan M, Tokarev A, Hsu D, Slike BM, Sacdalan C, et al. Cerebrospinal fluid CD4+ T cell infection in humans and macaques during acute HIV-1 and SHIV infection. PLoS Pathog. 2021;17(12):e1010105. doi: 10.1371/journal.ppat.1010105 34874976 PMC8683024

[55] Schnell G, Joseph S, Spudich S, Price RW, Swanstrom R. HIV-1 replication in the central nervous system occurs in two distinct cell types. PLoS Pathog. 2011;7(10):e1002286. doi: 10.1371/journal.ppat.1002286 22007152 PMC3188520

[56] Sturdevant CB, Joseph SB, Schnell G, Price RW, Swanstrom R, Spudich S. Compartmentalized replication of R5 T cell-tropic HIV-1 in the central nervous system early in the course of infection. PLoS Pathog. 2015;11(3):e1004720. doi: 10.1371/journal.ppat.1004720 25811757 PMC4374811

[57] Zink MC, Brice AK, Kelly KM, Queen SE, Gama L, Li M, et al. Simian immunodeficiency virus-infected macaques treated with highly active antiretroviral therapy have reduced central nervous system viral replication and inflammation but persistence of viral DNA. J Infect Dis. 2010;202(1):161–70. doi: 10.1086/653213 20497048 PMC2880623

[58] Trunfio M, Caballero G, Gomez-Moreno V, Mallal SA, Wanjalla CN, Jones A, et al. Central Nervous System T-cell immune architecture, and not HIV burden, tracks with cognition under long-term viral suppression. PLoS Pathog. 2026;22(6):e1014351. doi: 10.1371/journal.ppat.1014351 42296122 PMC13286276

[59] Tang Y, Xie L, Shabangu CS, Li D, da Silva Prates G, Manickam A, et al. Persistent type I interferon signaling within the brain of people with HIV on ART with cognitive impairment. PLoS Pathog. 2025;21(8):e1013411. doi: 10.1371/journal.ppat.1013411 40833988 PMC12367146

[60] Wilson AM, Jacobs MM, Lambert TY, Valada A, Meloni G, Gilmore E, et al. Transcriptional impacts of substance use disorder and HIV on human ventral midbrain neurons and microglia. Nat Commun. 2025;16(1):9123. doi: 10.1038/s41467-025-64193-5 41087367 PMC12521487

[61] Yadav A, Collman RG. CNS inflammation and macrophage/microglial biology associated with HIV-1 infection. J Neuroimmune Pharmacol. 2009;4(4):430–47. doi: 10.1007/s11481-009-9174-2 19768553 PMC5935112

[62] Dos Reis RS, Ignatz LA, Selvam S, Wagner MCE, Gianella S, Chaillon A, et al. Microglial reactivity and nodule formation are associated with Synaptodendritic damage in the brains of people with HIV-1. Brain Pathol. 2026;36(3):e70064. doi: 10.1111/bpa.70064 41467330 PMC13051873

[63] Rose R, Gonzalez-Perez MP, Nolan DJ, Cross S, Lamers SL, Luzuriaga K. Ultradeep HIV-1 proviral envelope sequencing reveals complex population structure within and between brain and splenic tissues. J Virol. 2021;95(23):e0120221. doi: 10.1128/JVI.01202-21 34495695 PMC8577351

[64] Hyman BT, Phelps CH, Beach TG, Bigio EH, Cairns NJ, Carrillo MC, et al. National Institute on Aging-Alzheimer’s Association guidelines for the neuropathologic assessment of Alzheimer’s disease. Alzheimers Dement. 2012;8(1):1–13. doi: 10.1016/j.jalz.2011.10.007 22265587 PMC3266529

[65] Milanini B, Valcour V. Differentiating HIV-associated neurocognitive disorders from Alzheimer’s disease: an emerging issue in geriatric neuroHIV. Curr HIV/AIDS Rep. 2017;14(4):123–32. doi: 10.1007/s11904-017-0361-0 28779301 PMC5823609

[66] Cserhati MF, Pandey S, Beaudoin JJ, Baccaglini L, Guda C, Fox HS. The National NeuroAIDS Tissue Consortium (NNTC) Database: an integrated database for HIV-related studies. Database. 2015;2015. doi: 10.1093/database/bav074 PMC4520230 26228431

[67] Plaza-Jennings AL, Valada A, O’Shea C, Iskhakova M, Hu B, Javidfar B, et al. HIV integration in the human brain is linked to microglial activation and 3D genome remodeling. Mol Cell. 2022;82(24):4647-4663.e8. doi: 10.1016/j.molcel.2022.11.016 36525955 PMC9831062

[68] Ament SA, Campbell RR, Lobo MK, Receveur JP, Agrawal K, Borjabad A, et al. The single-cell opioid responses in the context of HIV (SCORCH) consortium. Mol Psychiatry. 2024;29(12):3950–61. doi: 10.1038/s41380-024-02620-7 38879719 PMC11609103

[69] Cohen RA, Harezlak J, Gongvatana A, Buchthal S, Schifitto G, Clark U, et al. Cerebral metabolite abnormalities in human immunodeficiency virus are associated with cortical and subcortical volumes. J Neurovirol. 2010; 16 (6): 435–44. doi: 10.3109/13550284.2010.520817 20961212 PMC4560459

[70] Neuen-Jacob E, Arendt G, Wendtland B, Jacob B, Schneeweis M, Wechsler W. Frequency and topographical distribution of CD68-positive macrophages and HIV-1 core proteins in HIV-associated brain lesions. Clin Neuropathol. 1993;12(6):315–24. 8287624

[71] Zhou J, Jamal Eddine J, Byrnes SJ, Angelovich TA, Tumpach C, Churchill MJ. Regional heterogeneity of intact and defective HIV proviruses in the brain. Ann Neurol. 2023;94(4):726–39. 10.1002/ana.26750 PMC10914117 37493435

